# Supplementary material for: Cryo-EM structure of the vault from human brain reveals symmetry mismatch at its caps
Source: Structure. Author manuscript; Available in PMC 2026 Mar 17. (PMC7618893; doi:10.1016/j.str.2025.07.014)
Supplement: Document S1 [file EMS212831-supplement-Document_S1.pdf]

Structure, Volume *33*

Supplemental Information

Cryo-EM structure of the vault from human brain reveals symmetry mismatch at its caps

Sofia Lövestam and Sjors H.W. Scheres

Supplemental Information

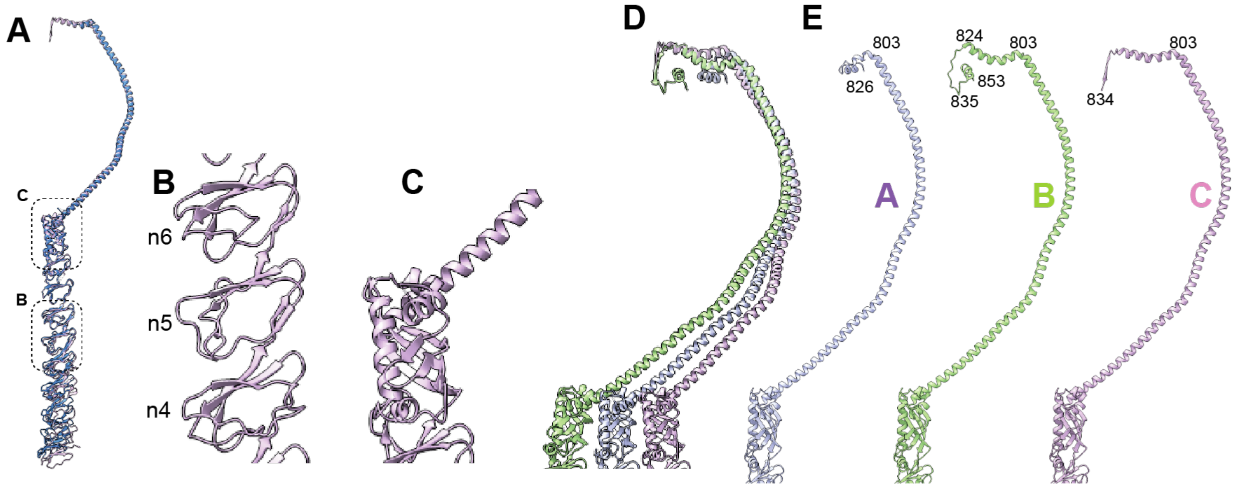

Figure S1: Structure of the Major Vault Protein subunit, related to Figure 2. **A** Major Vault Protein (MVP) from our cryo-EM structure (purple) overlaid with previously solved MVP structure (PDB:4hl8 in blue)[4]. **B** Close-up of n4, n5 and n6 repeat regions of the MVP **C** Close-up of the shoulder domain **D** Close-up of three different arrangements of the carboxy-terminus of the vault monomers where A is shown in purple, B in green and C in pink. **E** Separate views of the carboxy-terminus of monomers A-C.

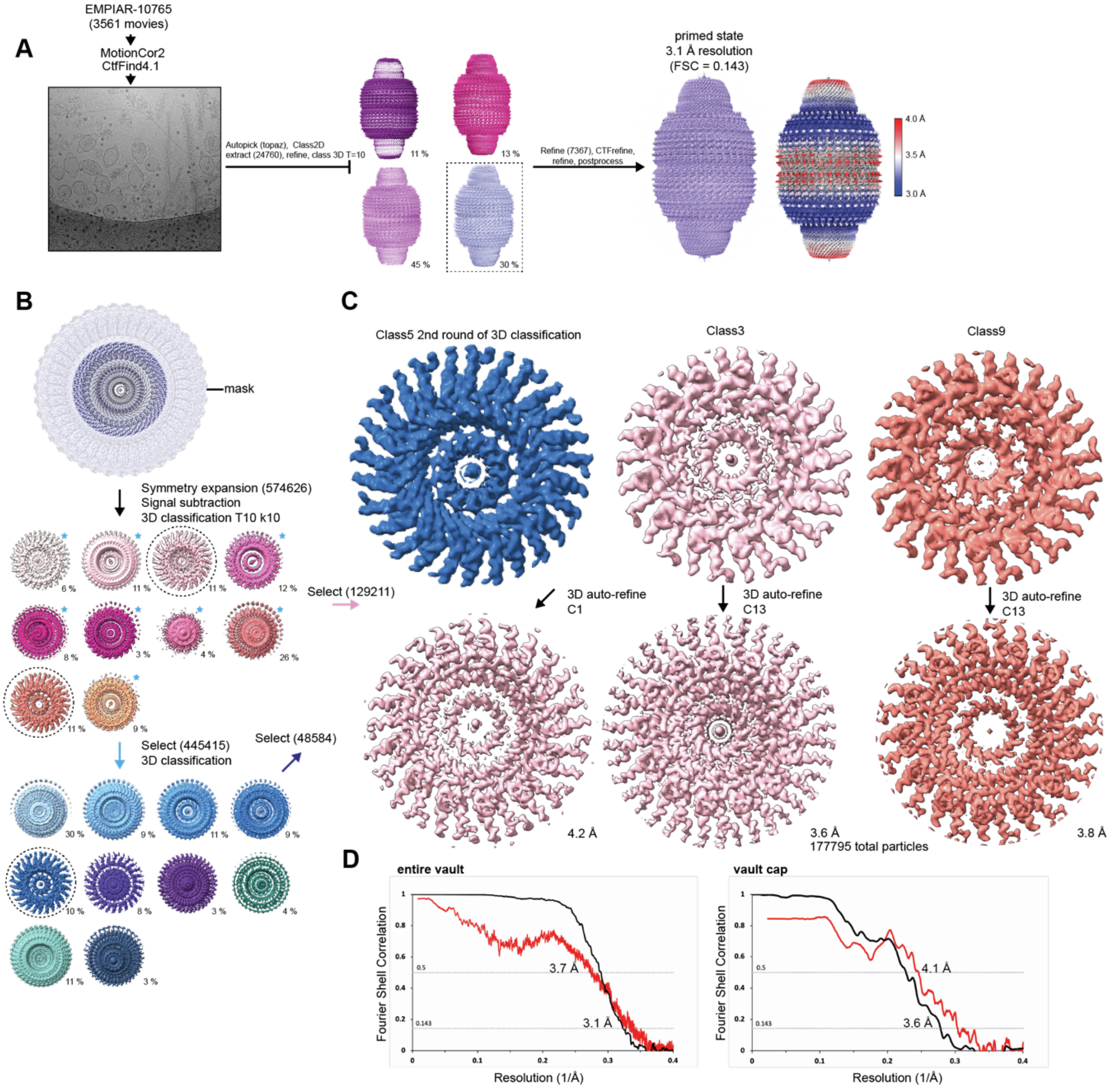

Figure S2: Cryo-EM data processing of the major vault protein, related to Figure 1.
A Cryo-EM micrographs were imported, corrected for motion and CTF parameters were estimated. Particles were autopicked using Topaz and particles were refined in RELION. 3D classification was used to select the best particles for CTF-refinement and for high-resolution 3D auto-refinement. Local resolution of the post-processed map is coloured from blue (3.0 Å) to red (4.0 Å). **B** Processing of the cap of the vault. A mask was made to include the cap of the vault and use partial signal subtraction for the remainder of the vault density. 3D classification using C13 symmetry was used to identify and select the best particles. **C** Selected particles were corrected for their first Euler angles by +/-360º/39 and used for 3D reconstruction in C13 symmetry, followed by standard RELION post-processing for resolution estimation and sharpening. **D** Fourier Shell Correlation curves are shown for two independently refined cryo-EM half maps (black) and for the final refined atomic model against the final cryo-EM map (red) for the D39-symmetric map of the entire vault and the C13 symmetric map of the cap.
